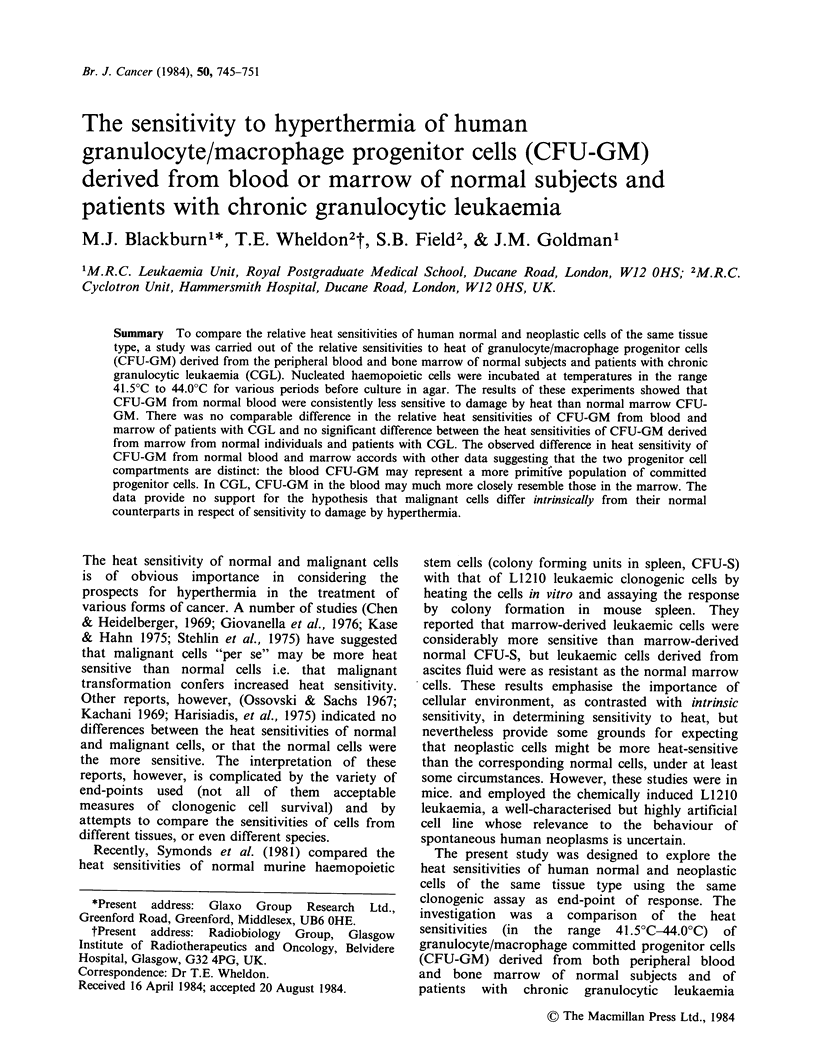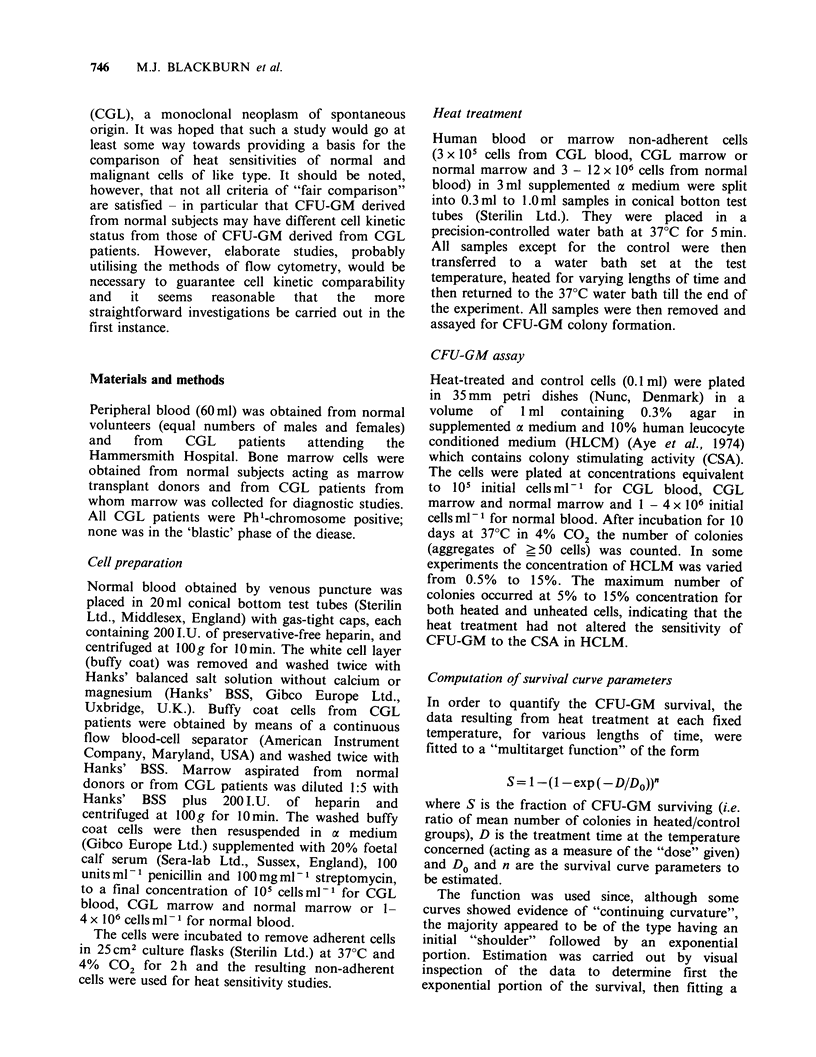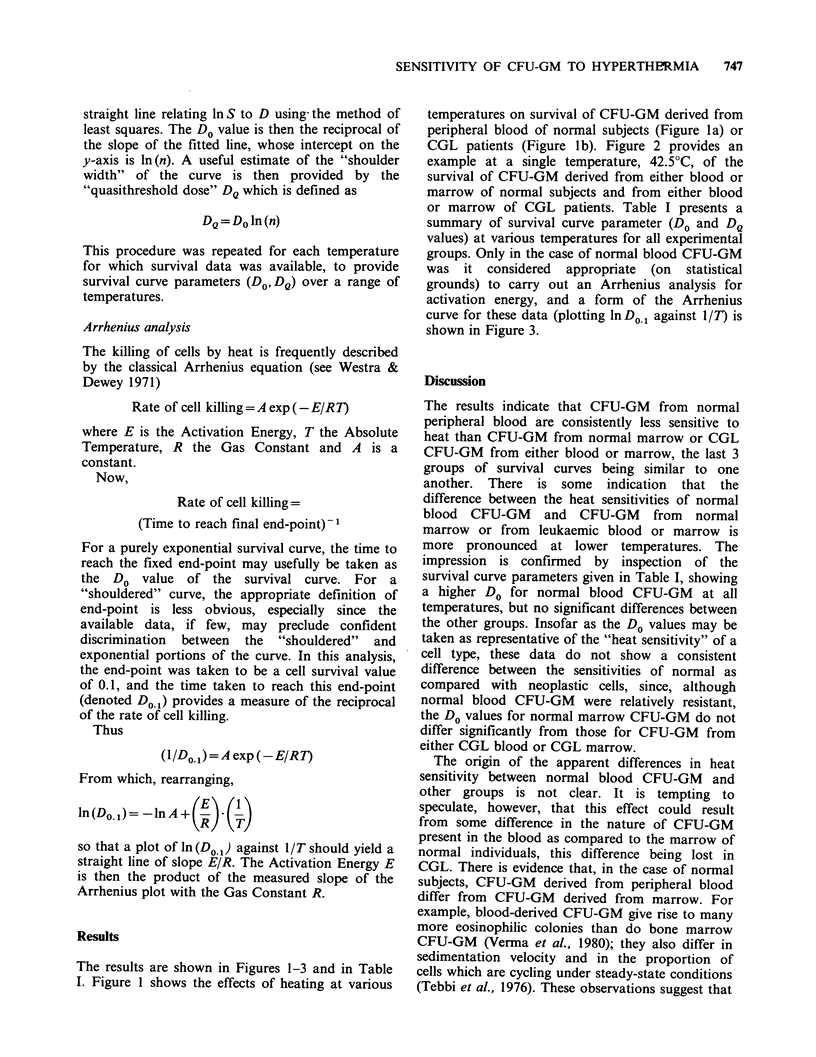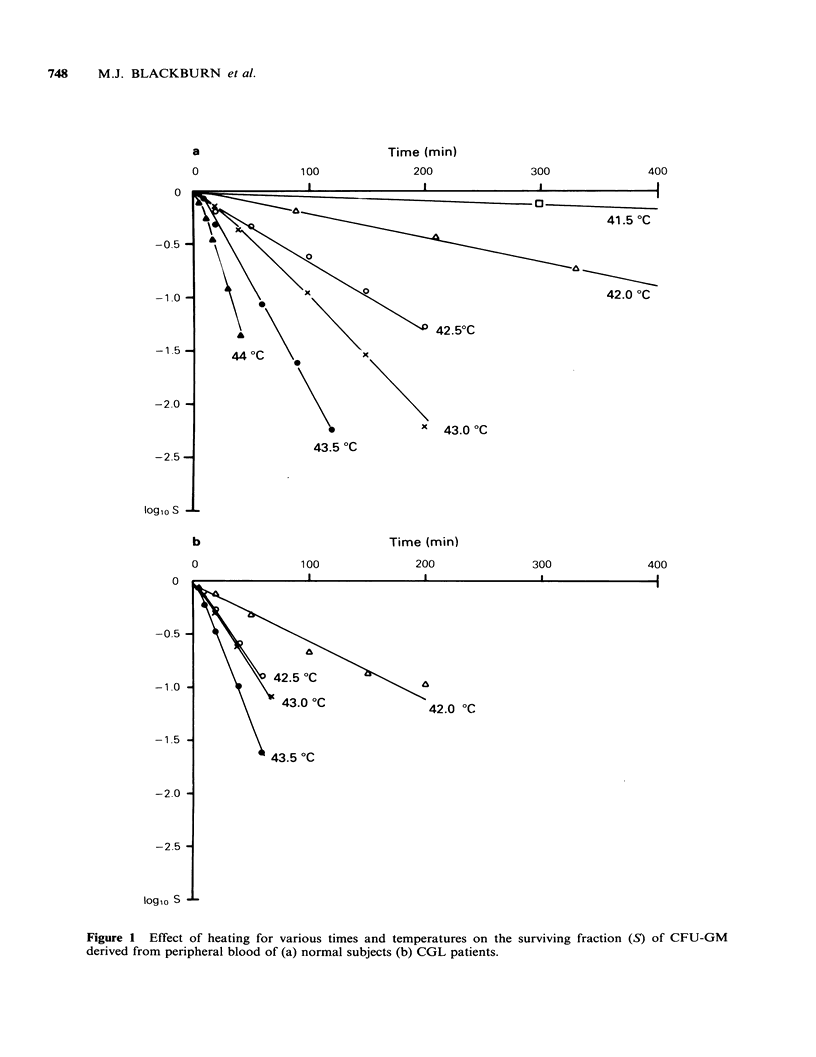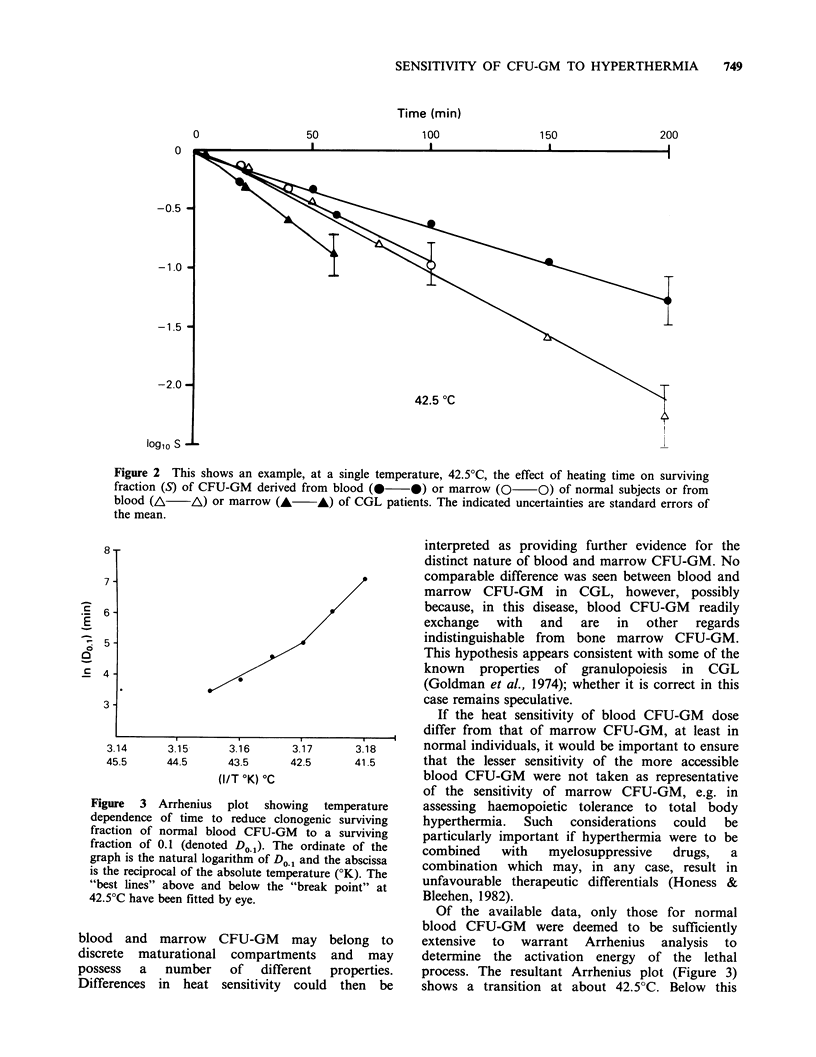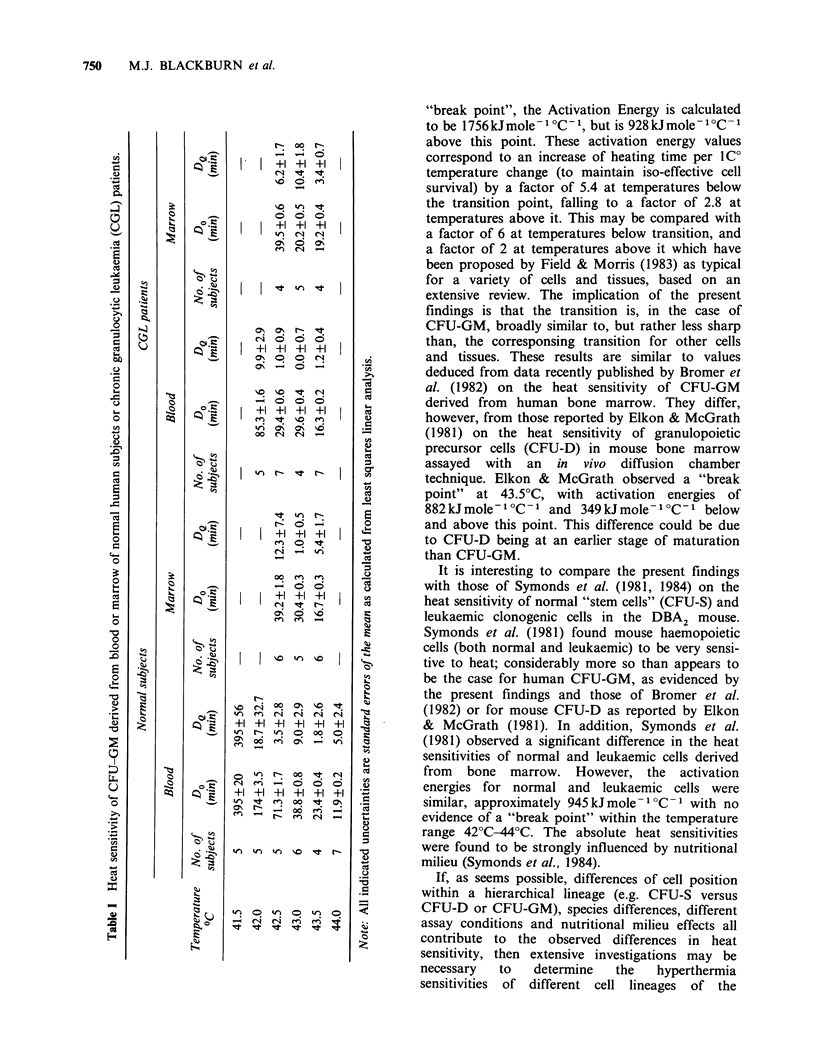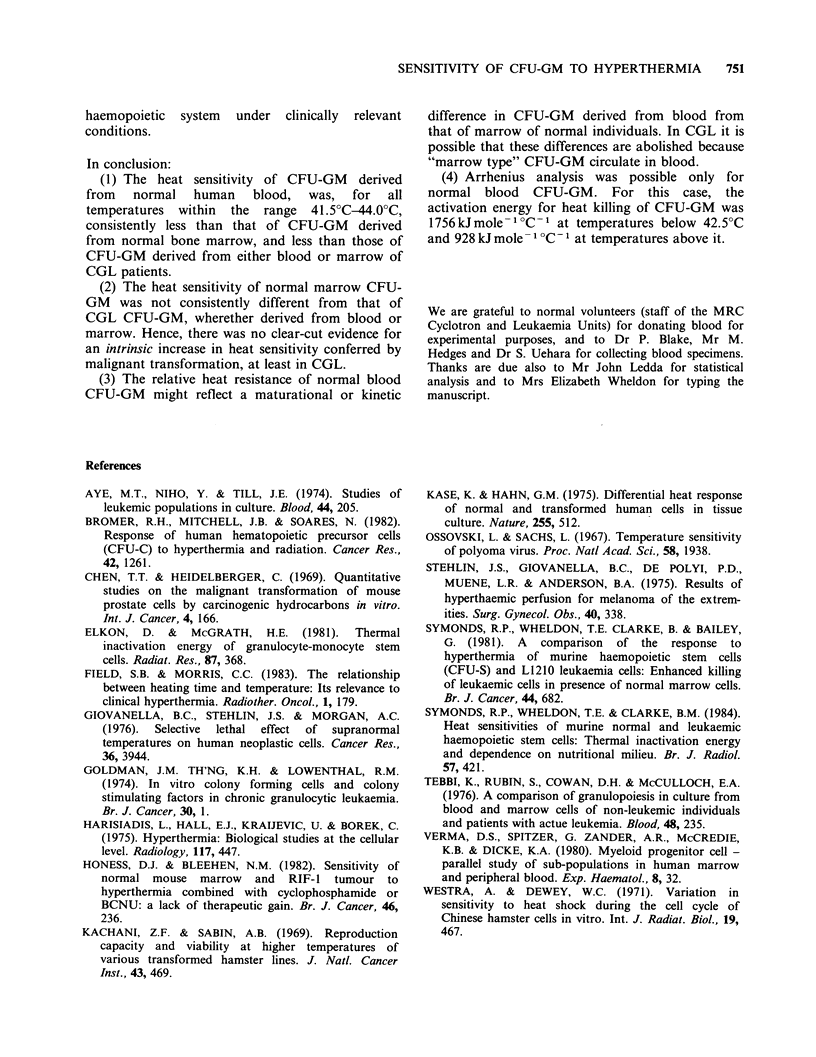# The sensitivity to hyperthermia of human granulocyte/macrophage progenitor cells (CFU-GM) derived from blood or marrow of normal subjects and patients with chronic granulocytic leukaemia.

**DOI:** 10.1038/bjc.1984.251

**Published:** 1984-12

**Authors:** M. J. Blackburn, T. E. Wheldon, S. B. Field, J. M. Goldman

## Abstract

To compare the relative heat sensitivities of human normal and neoplastic cells of the same tissue type, a study was carried out of the relative sensitivities to heat of granulocyte/macrophage progenitor cells (CFU-GM) derived from the peripheral blood and bone marrow of normal subjects and patients with chronic granulocytic leukaemia (CGL). Nucleated haemopoietic cells were incubated at temperatures in the range 41.5 degrees C to 44.0 degrees C for various periods before culture in agar. The results of these experiments showed that CFU-GM from normal blood were consistently less sensitive to damage by heat than normal marrow CFU-GM. There was no comparable difference in the relative heat sensitivities of CFU-GM from blood and marrow of patients with CGL and no significant difference between the heat sensitivities of CFU-GM derived from marrow from normal individuals and patients with CGL. The observed difference in heat sensitivity of CFU-GM from normal blood and marrow accords with other data suggesting that the two progenitor cell compartments are distinct: the blood CFU-GM may represent a more primitive population of committed progenitor cells. In CGL, CFU-GM in the blood may much more closely resemble those in the marrow. The data provide no support for the hypothesis that malignant cells differ intrinsically from their normal counterparts in respect of sensitivity to damage by hyperthermia.


Br. J. Cancer (1984), **50**, 745–751

### The sensitivity to hyperthermia of human granulocyte/macrophage progenitor cells (CFU-GM) derived from blood or marrow of normal subjects and patients with chronic granulocytic leukaemia

M.J. Blackburn[1*], T.E. Wheldon[2†], S.B. Field[2], & J.M. Goldman[1]

[1]*M.R.C. Leukaemia Unit, Royal Postgraduate Medical School, Ducane Road, London, W12 0HS;* [2]*M.R.C. Cyclotron Unit, Hammersmith Hospital, Ducane Road, London, W12 0HS, UK.*

**Summary** To compare the relative heat sensitivities of human normal and neoplastic cells of the same tissue type, a study was carried out of the relative sensitivities to heat of granulocyte/macrophage progenitor cells (CFU-GM) derived from the peripheral blood and bone marrow of normal subjects and patients with chronic granulocytic leukaemia (CGL). Nucleated haemopoietic cells were incubated at temperatures in the range 41.5°C to 44.0°C for various periods before culture in agar. The results of these experiments showed that CFU-GM from normal blood were consistently less sensitive to damage by heat than normal marrow CFU-GM. There was no comparable difference in the relative heat sensitivities of CFU-GM from blood and marrow of patients with CGL and no significant difference between the heat sensitivities of CFU-GM derived from marrow from normal individuals and patients with CGL. The observed difference in heat sensitivity of CFU-GM from normal blood and marrow accords with other data suggesting that the two progenitor cell compartments are distinct: the blood CFU-GM may represent a more primitive population of committed progenitor cells. In CGL, CFU-GM in the blood may much more closely resemble those in the marrow. The data provide no support for the hypothesis that malignant cells differ *intrinsically* from their normal counterparts in respect of sensitivity to damage by hyperthermia.

The heat sensitivity of normal and malignant cells is of obvious importance in considering the prospects for hyperthermia in the treatment of various forms of cancer. A number of studies (Chen & Heidelberger, 1969; Giovanella *et al.*, 1976; Kase & Hahn 1975; Stehlin *et al.*, 1975) have suggested that malignant cells "per se" may be more heat sensitive than normal cells i.e. that malignant transformation confers increased heat sensitivity. Other reports, however, (Ossovski & Sachs 1967; Kachani 1969; Harisiadis, *et al.*, 1975) indicated no differences between the heat sensitivities of normal and malignant cells, or that the normal cells were the more sensitive. The interpretation of these reports, however, is complicated by the variety of end-points used (not all of them acceptable measures of clonogenic cell survival) and by attempts to compare the sensitivities of cells from different tissues, or even different species.

Recently, Symonds *et al.* (1981) compared the heat sensitivities of normal murine haemopoietic stem cells (colony forming units in spleen, CFU-S) with that of L1210 leukaemic clonogenic cells by heating the cells *in vitro* and assaying the response by colony formation in mouse spleen. They reported that marrow-derived leukaemic cells were considerably more sensitive than marrow-derived normal CFU-S, but leukaemic cells derived from ascites fluid were as resistant as the normal marrow cells. These results emphasise the importance of cellular environment, as contrasted with *intrinsic* sensitivity, in determining sensitivity to heat, but nevertheless provide some grounds for expecting that neoplastic cells might be more heat-sensitive than the corresponding normal cells, under at least some circumstances. However, these studies were in mice. and employed the chemically induced L1210 leukaemia, a well-characterised but highly artificial cell line whose relevance to the behaviour of spontaneous human neoplasms is uncertain.

The present study was designed to explore the heat sensitivities of human normal and neoplastic cells of the same tissue type using the same clonogenic assay as end-point of response. The investigation was a comparison of the heat sensitivities (in the range 41.5°C–44.0°C) of granulocyte/macrophage committed progenitor cells (CFU-GM) derived from both peripheral blood and bone marrow of normal subjects and of patients with chronic granulocytic leukaemia

*Present address: Glaxo Group Research Ltd., Greenford Road, Greenford, Middlesex, UB6 0HE.

†Present address: Radiobiology Group, Glasgow Institute of Radiotherapeutics and Oncology, Belvidere Hospital, Glasgow, G32 4PG, UK.

Correspondence: Dr T.E. Wheldon.

Received 16 April 1984; accepted 20 August 1984.

© The Macmillan Press Ltd., 1984

(CGL), a monoclonal neoplasm of spontaneous origin. It was hoped that such a study would go at least some way towards providing a basis for the comparison of heat sensitivities of normal and malignant cells of like type. It should be noted, however, that not all criteria of "fair comparison" are satisfied – in particular that CFU-GM derived from normal subjects may have different cell kinetic status from those of CFU-GM derived from CGL patients. However, elaborate studies, probably utilising the methods of flow cytometry, would be necessary to guarantee cell kinetic comparability and it seems reasonable that the more straightforward investigations be carried out in the first instance.

### Materials and methods

Peripheral blood (60 ml) was obtained from normal volunteers (equal numbers of males and females) and from CGL patients attending the Hammersmith Hospital. Bone marrow cells were obtained from normal subjects acting as marrow transplant donors and from CGL patients from whom marrow was collected for diagnostic studies. All CGL patients were $Ph^1$-chromosome positive; none was in the 'blastic' phase of the diease.

### *Cell preparation*

Normal blood obtained by venous puncture was placed in 20 ml conical bottom test tubes (Sterilin Ltd., Middlesex, England) with gas-tight caps, each containing 200 I.U. of preservative-free heparin, and centrifuged at 100 *g* for 10 min. The white cell layer (buffy coat) was removed and washed twice with Hanks' balanced salt solution without calcium or magnesium (Hanks' BSS, Gibco Europe Ltd., Uxbridge, U.K.). Buffy coat cells from CGL patients were obtained by means of a continuous flow blood-cell separator (American Instrument Company, Maryland, USA) and washed twice with Hanks' BSS. Marrow aspirated from normal donors or from CGL patients was diluted 1:5 with Hanks' BSS plus 200 I.U. of heparin and centrifuged at 100 *g* for 10 min. The washed buffy coat cells were then resuspended in $\alpha$ medium (Gibco Europe Ltd.) supplemented with 20% foetal calf serum (Sera-lab Ltd., Sussex, England), 100 units $ml^{-1}$ penicillin and 100 mg $ml^{-1}$ streptomycin, to a final concentration of $10^5$ cells $ml^{-1}$ for CGL blood, CGL marrow and normal marrow or 1–$4 \times 10^6$ cells $ml^{-1}$ for normal blood.

The cells were incubated to remove adherent cells in 25 $cm^2$ culture flasks (Sterilin Ltd.) at 37°C and 4% $CO_2$ for 2 h and the resulting non-adherent cells were used for heat sensitivity studies.

### *Heat treatment*

Human blood or marrow non-adherent cells ($3 \times 10^5$ cells from CGL blood, CGL marrow or normal marrow and 3 – $12 \times 10^6$ cells from normal blood) in 3 ml supplemented $\alpha$ medium were split into 0.3 ml to 1.0 ml samples in conical botton test tubes (Sterilin Ltd.). They were placed in a precision-controlled water bath at 37°C for 5 min. All samples except for the control were then transferred to a water bath set at the test temperature, heated for varying lengths of time and then returned to the 37°C water bath till the end of the experiment. All samples were then removed and assayed for CFU-GM colony formation.

### *CFU-GM assay*

Heat-treated and control cells (0.1 ml) were plated in 35 mm petri dishes (Nunc, Denmark) in a volume of 1 ml containing 0.3% agar in supplemented $\alpha$ medium and 10% human leucocyte conditioned medium (HLCM) (Aye *et al.*, 1974) which contains colony stimulating activity (CSA). The cells were plated at concentrations equivalent to $10^5$ initial cells $ml^{-1}$ for CGL blood, CGL marrow and normal marrow and 1 – $4 \times 10^6$ initial cells $ml^{-1}$ for normal blood. After incubation for 10 days at 37°C in 4% $CO_2$ the number of colonies (aggregates of $\geqq 50$ cells) was counted. In some experiments the concentration of HCLM was varied from 0.5% to 15%. The maximum number of colonies occurred at 5% to 15% concentration for both heated and unheated cells, indicating that the heat treatment had not altered the sensitivity of CFU-GM to the CSA in HCLM.

### *Computation of survival curve parameters*

In order to quantify the CFU-GM survival, the data resulting from heat treatment at each fixed temperature, for various lengths of time, were fitted to a "multitarget function" of the form

$$S = 1 - (1 - \exp(-D/D_0))^n$$

where $S$ is the fraction of CFU-GM surviving (*i.e.* ratio of mean number of colonies in heated/control groups), $D$ is the treatment time at the temperature concerned (acting as a measure of the "dose" given) and $D_0$ and $n$ are the survival curve parameters to be estimated.

The function was used since, although some curves showed evidence of "continuing curvature", the majority appeared to be of the type having an initial "shoulder" followed by an exponential portion. Estimation was carried out by visual inspection of the data to determine first the exponential portion of the survival, then fitting a straight line relating $\ln S$ to $D$ using the method of least squares. The $D_0$ value is then the reciprocal of the slope of the fitted line, whose intercept on the $y$-axis is $\ln(n)$. A useful estimate of the "shoulder width" of the curve is then provided by the "quasithreshold dose" $D_Q$ which is defined as

$$D_Q = D_0 \ln(n)$$

This procedure was repeated for each temperature for which survival data was available, to provide survival curve parameters ($D_0, D_Q$) over a range of temperatures.

*Arrhenius analysis*

The killing of cells by heat is frequently described by the classical Arrhenius equation (see Westra & Dewey 1971)

$$\text{Rate of cell killing} = A \exp(-E/RT)$$

where $E$ is the Activation Energy, $T$ the Absolute Temperature, $R$ the Gas Constant and $A$ is a constant.

Now,

$$\text{Rate of cell killing} = (\text{Time to reach final end-point})^{-1}$$

For a purely exponential survival curve, the time to reach the fixed end-point may usefully be taken as the $D_0$ value of the survival curve. For a "shouldered" curve, the appropriate definition of end-point is less obvious, especially since the available data, if few, may preclude confident discrimination between the "shouldered" and exponential portions of the curve. In this analysis, the end-point was taken to be a cell survival value of 0.1, and the time taken to reach this end-point (denoted $D_{0.1}$) provides a measure of the reciprocal of the rate of cell killing.

Thus

$$(1/D_{0.1}) = A \exp(-E/RT)$$

From which, rearranging,

$$\ln(D_{0.1}) = -\ln A + \left(\frac{E}{R}\right) \cdot \left(\frac{1}{T}\right)$$

so that a plot of $\ln(D_{0.1})$ against $1/T$ should yield a straight line of slope $E/R$. The Activation Energy $E$ is then the product of the measured slope of the Arrhenius plot with the Gas Constant $R$.

### Results

The results are shown in Figures 1–3 and in Table I. Figure 1 shows the effects of heating at various temperatures on survival of CFU-GM derived from peripheral blood of normal subjects (Figure 1a) or CGL patients (Figure 1b). Figure 2 provides an example at a single temperature, 42.5°C, of the survival of CFU-GM derived from either blood or marrow of normal subjects and from either blood or marrow of CGL patients. Table I presents a summary of survival curve parameter ($D_0$ and $D_Q$ values) at various temperatures for all experimental groups. Only in the case of normal blood CFU-GM was it considered appropriate (on statistical grounds) to carry out an Arrhenius analysis for activation energy, and a form of the Arrhenius curve for these data (plotting $\ln D_{0.1}$ against $1/T$) is shown in Figure 3.

### Discussion

The results indicate that CFU-GM from normal peripheral blood are consistently less sensitive to heat than CFU-GM from normal marrow or CGL CFU-GM from either blood or marrow, the last 3 groups of survival curves being similar to one another. There is some indication that the difference between the heat sensitivities of normal blood CFU-GM and CFU-GM from normal marrow or from leukaemic blood or marrow is more pronounced at lower temperatures. The impression is confirmed by inspection of the survival curve parameters given in Table I, showing a higher $D_0$ for normal blood CFU-GM at all temperatures, but no significant differences between the other groups. Insofar as the $D_0$ values may be taken as representative of the "heat sensitivity" of a cell type, these data do not show a consistent difference between the sensitivities of normal as compared with neoplastic cells, since, although normal blood CFU-GM were relatively resistant, the $D_0$ values for normal marrow CFU-GM do not differ significantly from those for CFU-GM from either CGL blood or CGL marrow.

The origin of the apparent differences in heat sensitivity between normal blood CFU-GM and other groups is not clear. It is tempting to speculate, however, that this effect could result from some difference in the nature of CFU-GM present in the blood as compared to the marrow of normal individuals, this difference being lost in CGL. There is evidence that, in the case of normal subjects, CFU-GM derived from peripheral blood differ from CFU-GM derived from marrow. For example, blood-derived CFU-GM give rise to many more eosinophilic colonies than do bone marrow CFU-GM (Verma *et al.*, 1980); they also differ in sedimentation velocity and in the proportion of cells which are cycling under steady-state conditions (Tebbi *et al.*, 1976). These observations suggest that

**Figure 1** Effect of heating for various times and temperatures on the surviving fraction (*S*) of CFU-GM derived from peripheral blood of (a) normal subjects (b) CGL patients.

**Figure 2** This shows an example, at a single temperature, 42.5°C, the effect of heating time on surviving fraction ($S$) of CFU-GM derived from blood (●——●) or marrow (○——○) of normal subjects or from blood (△——△) or marrow (▲——▲) of CGL patients. The indicated uncertainties are standard errors of the mean.

**Figure 3** Arrhenius plot showing temperature dependence of time to reduce clonogenic surviving fraction of normal blood CFU-GM to a surviving fraction of 0.1 (denoted $D_{0.1}$). The ordinate of the graph is the natural logarithm of $D_{0.1}$ and the abscissa is the reciprocal of the absolute temperature (°K). The "best lines" above and below the "break point" at 42.5°C have been fitted by eye.

blood and marrow CFU-GM may belong to discrete maturational compartments and may possess a number of different properties. Differences in heat sensitivity could then be interpreted as providing further evidence for the distinct nature of blood and marrow CFU-GM. No comparable difference was seen between blood and marrow CFU-GM in CGL, however, possibly because, in this disease, blood CFU-GM readily exchange with and are in other regards indistinguishable from bone marrow CFU-GM. This hypothesis appears consistent with some of the known properties of granulopoiesis in CGL (Goldman *et al.*, 1974); whether it is correct in this case remains speculative.

If the heat sensitivity of blood CFU-GM dose differ from that of marrow CFU-GM, at least in normal individuals, it would be important to ensure that the lesser sensitivity of the more accessible blood CFU-GM were not taken as representative of the sensitivity of marrow CFU-GM, e.g. in assessing haemopoietic tolerance to total body hyperthermia. Such considerations could be particularly important if hyperthermia were to be combined with myelosuppressive drugs, a combination which may, in any case, result in unfavourable therapeutic differentials (Honess & Bleehen, 1982).

Of the available data, only those for normal blood CFU-GM were deemed to be sufficiently extensive to warrant Arrhenius analysis to determine the activation energy of the lethal process. The resultant Arrhenius plot (Figure 3) shows a transition at about 42.5°C. Below this

**Table I** Heat sensitivity of CFU-GM derived from blood or marrow of normal human subjects or chronic granulocytic leukaemia (CGL) patients.

| | Normal subjects | | | | | | CGL patients | | | | | |
|---|---|---|---|---|---|---|---|---|---|---|---|---|
| | Blood | | | Marrow | | | Blood | | | Marrow | | |
| *Temperature* °C | *No. of subjects* | $D_0$ *(min)* | $D_Q$ *(min)* | *No. of subjects* | $D_0$ *(min)* | $D_Q$ *(min)* | *No. of subjects* | $D_0$ *(min)* | $D_Q$ *(min)* | *No. of subjects* | $D_0$ *(min)* | $D_Q$ *(min)* |
| 41.5 | 5 | 395±20 | 395±56 | — | — | — | — | — | — | — | — | — |
| 42.0 | 5 | 174±3.5 | 18.7±32.7 | — | — | — | 5 | 85.3±1.6 | 9.9±2.9 | — | — | — |
| 42.5 | 5 | 71.3±1.7 | 3.5±2.8 | 6 | 39.2±1.8 | 12.3±7.4 | 7 | 29.4±0.6 | 1.0±0.9 | 4 | 39.5±0.6 | 6.2±1.7 |
| 43.0 | 6 | 38.8±0.8 | 9.0±2.9 | 5 | 30.4±0.3 | 1.0±0.5 | 4 | 29.6±0.4 | 0.0±0.7 | 5 | 20.2±0.5 | 10.4±1.8 |
| 43.5 | 4 | 23.4±0.4 | 1.8±2.6 | 6 | 16.7±0.3 | 5.4±1.7 | 7 | 16.3±0.2 | 1.2±0.4 | 4 | 19.2±0.4 | 3.4±0.7 |
| 44.0 | 7 | 11.9±0.2 | 5.0±2.4 | — | — | — | — | — | — | — | — | — |

*Note:* All indicated uncertainties are *standard errors of the mean* as calculated from least squares linear analysis.

"break point", the Activation Energy is calculated to be 1756 kJ mole$^{-1}$ °C$^{-1}$, but is 928 kJ mole$^{-1}$ °C$^{-1}$ above this point. These activation energy values correspond to an increase of heating time per 1C° temperature change (to maintain iso-effective cell survival) by a factor of 5.4 at temperatures below the transition point, falling to a factor of 2.8 at temperatures above it. This may be compared with a factor of 6 at temperatures below transition, and a factor of 2 at temperatures above it which have been proposed by Field & Morris (1983) as typical for a variety of cells and tissues, based on an extensive review. The implication of the present findings is that the transition is, in the case of CFU-GM, broadly similar to, but rather less sharp than, the corresponsing transition for other cells and tissues. These results are similar to values deduced from data recently published by Bromer *et al.* (1982) on the heat sensitivity of CFU-GM derived from human bone marrow. They differ, however, from those reported by Elkon & McGrath (1981) on the heat sensitivity of granulopoietic precursor cells (CFU-D) in mouse bone marrow assayed with an *in vivo* diffusion chamber technique. Elkon & McGrath observed a "break point" at 43.5°C, with activation energies of 882 kJ mole$^{-1}$ °C$^{-1}$ and 349 kJ mole$^{-1}$ °C$^{-1}$ below and above this point. This difference could be due to CFU-D being at an earlier stage of maturation than CFU-GM.

It is interesting to compare the present findings with those of Symonds *et al.* (1981, 1984) on the heat sensitivity of normal "stem cells" (CFU-S) and leukaemic clonogenic cells in the $DBA_2$ mouse. Symonds *et al.* (1981) found mouse haemopoietic cells (both normal and leukaemic) to be very sensitive to heat; considerably more so than appears to be the case for human CFU-GM, as evidenced by the present findings and those of Bromer *et al.* (1982) or for mouse CFU-D as reported by Elkon & McGrath (1981). In addition, Symonds *et al.* (1981) observed a significant difference in the heat sensitivities of normal and leukaemic cells derived from bone marrow. However, the activation energies for normal and leukaemic cells were similar, approximately 945 kJ mole$^{-1}$ °C$^{-1}$ with no evidence of a "break point" within the temperature range 42°C–44°C. The absolute heat sensitivities were found to be strongly influenced by nutritional milieu (Symonds *et al.*, 1984).

If, as seems possible, differences of cell position within a hierarchical lineage (e.g. CFU-S versus CFU-D or CFU-GM), species differences, different assay conditions and nutritional milieu effects all contribute to the observed differences in heat sensitivity, then extensive investigations may be necessary to determine the hyperthermia sensitivities of different cell lineages of the haemopoietic system under clinically relevant conditions.

In conclusion:

(1) The heat sensitivity of CFU-GM derived from normal human blood, was, for all temperatures within the range 41.5°C–44.0°C, consistently less than that of CFU-GM derived from normal bone marrow, and less than those of CFU-GM derived from either blood or marrow of CGL patients.

(2) The heat sensitivity of normal marrow CFU-GM was not consistently different from that of CGL CFU-GM, wherether derived from blood or marrow. Hence, there was no clear-cut evidence for an *intrinsic* increase in heat sensitivity conferred by malignant transformation, at least in CGL.

(3) The relative heat resistance of normal blood CFU-GM might reflect a maturational or kinetic difference in CFU-GM derived from blood from that of marrow of normal individuals. In CGL it is possible that these differences are abolished because "marrow type" CFU-GM circulate in blood.

(4) Arrhenius analysis was possible only for normal blood CFU-GM. For this case, the activation energy for heat killing of CFU-GM was 1756 kJ mole$^{-1}$ °C$^{-1}$ at temperatures below 42.5°C and 928 kJ mole$^{-1}$ °C$^{-1}$ at temperatures above it.

We are grateful to normal volunteers (staff of the MRC Cyclotron and Leukaemia Units) for donating blood for experimental purposes, and to Dr P. Blake, Mr M. Hedges and Dr S. Uehara for collecting blood specimens. Thanks are due also to Mr John Ledda for statistical analysis and to Mrs Elizabeth Wheldon for typing the manuscript.

**References**

AYE, M.T., NIHO, Y. & TILL, J.E. (1974). Studies of leukemic populations in culture. *Blood*, **44**, 205.

BROMER, R.H., MITCHELL, J.B. & SOARES, N. (1982). Response of human hematopoietic precursor cells (CFU-C) to hyperthermia and radiation. *Cancer Res.*, **42**, 1261.

CHEN, T.T. & HEIDELBERGER, C. (1969). Quantitative studies on the malignant transformation of mouse prostate cells by carcinogenic hydrocarbons *in vitro*. *Int. J. Cancer*, **4**, 166.

ELKON, D. & MCGRATH, H.E. (1981). Thermal inactivation energy of granulocyte-monocyte stem cells. *Radiat. Res.*, **87**, 368.

FIELD, S.B. & MORRIS, C.C. (1983). The relationship between heating time and temperature: Its relevance to clinical hyperthermia. *Radiother. Oncol.*, **1**, 179.

GIOVANELLA, B.C., STEHLIN, J.S. & MORGAN, A.C. (1976). Selective lethal effect of supranormal temperatures on human neoplastic cells. *Cancer Res.*, **36**, 3944.

GOLDMAN, J.M. TH'NG, K.H. & LOWENTHAL, R.M. (1974). In vitro colony forming cells and colony stimulating factors in chronic granulocytic leukaemia. *Br. J. Cancer*, **30**, 1.

HARISIADIS, L., HALL, E.J., KRAIJEVIC, U. & BOREK, C. (1975). Hyperthermia: Biological studies at the cellular level. *Radiology*, **117**, 447.

HONESS, D.J. & BLEEHEN, N.M. (1982). Sensitivity of normal mouse marrow and RIF-1 tumour to hyperthermia combined with cyclophosphamide or BCNU: a lack of therapeutic gain. *Br. J. Cancer*, **46**, 236.

KACHANI, Z.F. & SABIN, A.B. (1969). Reproduction capacity and viability at higher temperatures of various transformed hamster lines. *J. Natl. Cancer Inst.*, **43**, 469.

KASE, K. & HAHN, G.M. (1975). Differential heat response of normal and transformed human cells in tissue culture. *Nature*, **255**, 512.

OSSOVSKI, L. & SACHS, L. (1967). Temperature sensitivity of polyoma virus. *Proc. Natl Acad. Sci.*, **58**, 1938.

STEHLIN, J.S., GIOVANELLA, B.C., DE POLYI, P.D., MUENE, L.R. & ANDERSON, B.A. (1975). Results of hyperthaemic perfusion for melanoma of the extremities. *Surg. Gynecol. Obs.*, **40**, 338.

SYMONDS, R.P., WHELDON, T.E. CLARKE, B. & BAILEY, G. (1981). A comparison of the response to hyperthermia of murine haemopoietic stem cells (CFU-S) and L1210 leukaemia cells: Enhanced killing of leukaemic cells in presence of normal marrow cells. *Br. J. Cancer*, **44**, 682.

SYMONDS, R.P., WHELDON, T.E. & CLARKE, B.M. (1984). Heat sensitivities of murine normal and leukaemic haemopoietic stem cells: Thermal inactivation energy and dependence on nutritional milieu. *Br. J. Radiol.* **57**, 421.

TEBBI, K., RUBIN, S., COWAN, D.H. & MCCULLOCH, E.A. (1976). A comparison of granulopoiesis in culture from blood and marrow cells of non-leukemic individuals and patients with actue leukemia. *Blood*, **48**, 235.

VERMA, D.S., SPITZER, G. ZANDER, A.R., MCCREDIE, K.B. & DICKE, K.A. (1980). Myeloid progenitor cell – parallel study of sub-populations in human marrow and peripheral blood. *Exp. Haematol.*, **8**, 32.

WESTRA, A. & DEWEY, W.C. (1971). Variation in sensitivity to heat shock during the cell cycle of Chinese hamster cells in vitro. Int. *J. Radiat. Biol.*, **19**, 467.